# Single CD271 marker isolates mesenchymal stem cells from human dental pulp

**DOI:** 10.1038/ijos.2015.29

**Published:** 2015-09-18

**Authors:** Ruth Alvarez, Hye-Lim Lee, Christine Hong, Cun-Yu Wang

**Affiliations:** 1 Division of Oral Biology and Medicine, School of Dentistry, University of California at Los Angeles, Los Angeles, USA; 2 Section of Orthodontics, School of Dentistry, University of California at Los Angeles, Los Angeles, USA; 3 Department of Bioengineering, Henry Samueli School of Engineering and Applied Science, University of California at Los Angeles, Los Angeles, USA

**Keywords:** dental mesenchymal stem cells, odontogenic differentiation, cell surface markers, dental pulp, fluorescence-activated cell sorting

## Abstract

Mesenchymal stem cells (MSCs) are a promising tool in regenerative medicine due to their capacity to differentiate into multiple lineages. In addition to MSCs isolated from bone marrow (BMSCs), adult MSCs are isolated from craniofacial tissues including dental pulp tissues (DPs) using various stem cell surface markers. However, there has been a lack of consensus on a set of surface makers that are reproducibly effective at isolating putative multipotent dental mesenchymal stem cells (DMSCs). In this study, we used different combinations of surface markers (CD51/CD140α, CD271, and STRO-1/CD146) to isolate homogeneous populations of DMSCs from heterogeneous dental pulp cells (DPCs) obtained from DP and compared their capacity to undergo multilineage differentiation. Fluorescence-activated cell sorting revealed that 27.3% of DPCs were CD51^+^/CD140α^+^, 10.6% were CD271^+^, and 0.3% were STRO-1^+^/CD146^+^. Under odontogenic conditions, all three subsets of isolated DMSCs exhibited differentiation capacity into odontogenic lineages. Among these isolated subsets of DMSCs, CD271^+^ DMSCs demonstrated the greatest odontogenic potential. While all three combinations of surface markers in this study successfully isolated DMSCs from DPCs, the single CD271 marker presents the most effective stem cell surface marker for identification of DMSCs with high odontogenic potential. Isolated CD271^+^ DMSCs could potentially be utilized for future clinical applications in dentistry and regenerative medicine.

## Introduction

Embryonic stem cells (ESCs) have the ability to renew themselves and to differentiate into various specialized tissues, making them an attractive treatment modality in regenerative medicine.^1^ However, due to ethical concerns of isolating and using ESCs, great interest has focused on adult mesenchymal stem cells (MSCs), which are also characterized by their self-renewal and multipotent capacity.^1,2,3^ MSCs are believed to be responsible for growth, wound healing, and replacement of cells that are lost through daily wear and tear as well as pathological conditions, suggesting that they are important for tissue repair and maintenance.^1^

While MSCs derived from bone marrow (BMSCs) are the most widely recognized and studied, alternate sources for MSCs have been investigated due to the complications associated with harvesting BMSCs, such as pain, morbidity, and low cell number. Evidence has suggested that MSCs may be present in virtually any vascularized tissue in the body.^4,5^ In recent years, MSCs derived from craniofacial tissues including dental mesenchymal stem cells (DMSCs) have been identified as a putative alternative.^6^ Similar to BMSCs, these DMSCs are multipotent progenitor cells with: (1) the capability to self-renew and differentiate into a variety of cell types, (2) ease of isolation, and (3) lack of immunogenicity.^7^ When compared to BMSCs, DMSCs may be more advantageous for regenerating craniofacial defects because they are readily available without risk as DMSCs can be easily isolated from discarded tissues such as third molars.^8^ Approximately, 70% of dental patients have third molars that require extractions consequently providing an abundant source of DMSCs that can be cryopreserved for potential future use.^9^ These DMSCs can be obtained from dental pulp tissues (DPs), periodontal ligament, and apical papilla.^10^ Among these different types of DMSCs, DMSCs from DP are the most widely studied as similarities in biochemical pathways for odonto/osteogenic differentiation between BMSCs and DMSCs from DP have already been established.^11^

MSCs are a heterogeneous population of cells with varying magnitudes of differentiation potential among single clones of MSCs.^12^ It was documented that *in vitro* single cell cloning of human MSCs demonstrated that approximately 30% of the clonal cells were multipotent and thus “true” MSCs.^12^ Currently, numerous cell surface markers including STRO-1, CD29, CD44, CD73, CD90, CD105, CD106, and CD146 have been utilized to isolate homogenous and multipotent MSC populations.^10,13^ However, a consensus on surface markers to isolate DMSCs with high differentiation potential is lacking. As the future of successful craniofacial defect repair is dependent on the ability to isolate specific subsets of DMSCs with potent differentiation capacity into appropriate cell types, the identification of markers that isolate multipotent DMSCs effectively is critical. In this study, we assessed the effectiveness of different cell surface markers (CD51/CD140α, CD271, STRO-1/CD146) in isolating DMSCs from DP and examined odontogenic and chondrogenic potential of these isolated subsets of DMSCs.

## Materials and methods

### Cell isolation and culture

Primary dental pulp cells (DPCs) were isolated from DP of extracted adult third molars (IRB#13-000241-CR-00001) as previously described.^14^ DPCs were cultured in alpha modified Eagle's medium (α-MEM; Invitrogen, Carlsbad, CA, USA) containing 20% fetal bovine serum (FBS), non-essential amino acids, 100 units per mL penicillin, and 100 units per mL streptomycin, in a humidified 5% CO_2_ incubator at 37 °C (all reagents were from Invitrogen, Carlsbad, CA, USA). Media was changed every 2–3 days, and cells were passaged at 80%–90% confluency. DPCs at passages 4–8 were utilized in this study.

### Fluorescence-activated cell sorting

Expression of stem cell surface markers in DPCs was determined by fluorescence-activated cell sorting (FACS) analysis. The cells were detached using trypsin in 0.25% elhylene diamine tetraacetic acid (EDTA). After neutralization, single-cell suspensions were washed with phosphate-buffered saline supplemented with 2% FBS and 0.01% NaN_3_ (FACS buffer). Quantities of 1 × 10^5^ cells were incubated with the conjugated antibody for 20 min on ice in the dark. After washing, fluorescence intensity was measured on FACS Aria II cell sorter (BD Biosciences, San Jose, CA, USA). The following antibodies were used: Phycoerythrin (PE)-CD271 (Miltenyi Biotec, Auburn, CA, USA), Fluorescein Isothiocyanate (FITC)-CD90 (Biolegend, San Diego, CA, USA), allophycocyanin (APC)-CD106 (Biolegend, San Diego, CA, USA) or dual color combinations APC-STRO-1/PE-CD146 (Both from: Biolegend, San Diego, CA, USA), and PE-CD51 (Biolegend, San Diego, CA, USA)/APC-CD140α (BD Biosciences, San Jose, CA, USA). PE-IgG was used as a negative control.

### Induction of odontogenic differentiation

Sorted DMSCs were plated at 1 × 10^5^ cells per mL into 12-well plates. To induce differentiation into odontogenic lineages, sorted DMSCs were grown in odontogenic induction medium (OIM). OIM contains 90% minimum essential medium (α-MEM; Invitrogen, Carlsbad, CA, USA), 10% FBS (Invitrogen, Carlsbad, CA, USA), 50 μg·mL^−1^ ascorbic acid, 5 mmol·L^−1^ β-glycerolphosphate, and 100 nmol·L^−1^ dexamethasone (all from Sigma-Aldrich, St Louis, MO, USA). OIM was changed every 2–3 days. For alkaline phosphatase (ALP) staining, after odontogenic induction for 7 days, cells were fixed with 4% paraformaldehyde and incubated with a solution of 0.25% naphthol AS-BI phosphate and 0.75% Fast Blue BB (Sigma-Aldrich, St Louis, MO, USA) dissolved in 0.1 mol·L^−1^ tris(hydroxymethyl)aminomethane (Tris) buffer (pH 9.3). ALP activity assay was preformed using an ALP kit (Sigma-Aldrich, St Louis, MO, USA) according to the manufacturer's protocol and normalized based on protein concentrations. To detect mineralization potential, cells were induced for 2 weeks using OIM, fixed with 4% paraformaldehyde, and stained with 2% Alizarin red (Sigma-Aldrich, St Louis, MO, USA). For quantification, Alizarin red stain (ARS) was destained with 10% cetylprydiniumcholoride in 10 mmol·L^−1^ sodium phosphate for 30 min at room temperature. The optical absorbance was measured at 562 nm using a microplate reader with a standard calcium curve in the same solution. The final calcium level in each group was normalized with the total protein concentrations prepared from a duplicate plate.

### Induction of chondrogenic differentiation

Sorted DMSCs were plated at 1 × 10^5^ cells per mL into 12-well plates. To induce differentiation into chondrogenic lineages, sorted DMSCs were grown in chondrogenic induction medium containing α-MEM (Invitrogen, Carlsbad, CA, USA) supplemented with 10% FBS (Invitrogen, Carlsbad, CA, USA), 100 mmol·L^−1^ sodium pyruvate, 40 µg·mL^−1^ proline, 100 nmol·L^−1^ dexamethasone, 200 μmol·L^−1^ ascorbic acid (all from Sigma-Aldrich, St Louis, MO, USA), and 10 ng·mL^−1^ TGF-β (R&D systems, Minneapolis, MN, USA). Culture medium was changed every 2–3 days. After 4 weeks of differentiation *in vitro*, Alcian Blue Staining (Sigma-Aldrich, St Louis, MO, USA) was performed as previously described.^15^ For quantification, stained Alcian blue was eluted with 6 mol·L^−1^ guanidine HCl for 8 hours at room temperature. The optical absorbance was measured at 620 nm using a microplate reader.

### Quantitative real-time PCR

Total RNA was isolated from DMSCs using Trizol reagents (Invitrogen, Carlsbad, CA, USA). Two-µg aliquots of RNAs were synthesized using random hexamers and reverse transcriptase according to the manufacturer's protocol (Invitrogen, Carlsbad, Hercules, CA, USA). Real-time polymerase chain reaction (RT-PCR) performed using the QuantiTect SYBR Green PCR kit (Qiagen, Valencia, CA, USA) and the Icycler IQ Multi-color Real-time PCR Detection System (Bio-Rad, Hercules, CA, USA). The primers for RUNX2 (NM_001015051.3) were: forward, 5′-TGGTTACTGTCATGGCGGGTA-3′; reverse, 5′-TCTCAGATCGTTGAACCTTGCTA-3′. The primers for DLX5 (NM_005221.5) were: forward, 5′-GCTCTCAACCCCTACCAGTAT-3′; reverse, 5′-CTTTGGTTTGCCATTCACCATTC-3′. The primers for BGLAP (NM_199173.4) were: forward, 5′-AGCAAAGGTGCAGCCTTTGT-3′; reverse 5′-GCGCCTGGTCTCTTCACT-3′. The primers for DMP1 (NM_001079911.2) were: forward, 5′-GACAGCAAGGGTGACTCTCA-3′; reverse: 5′-AGATGACAGGTTGGCCTCTT-3′. The primers for DSPP (NM_014208.3) were: forward, 5′-TCAACAGCAAGAGAAATGGG-3′; reverse: 5′-TCGTCTTCATCCTCATCTGC-3′. The primers for SOX9 (NM_000346.3) were: forward, 5′-CACACAGCTCACTCGACCTT-3′; reverse, 5′-CAAAGGGAATTCTGGTTGGT-3′. Primers were designed with GenScript Real-time PCR (TaqMan) Primer Design program. The cycling conditions were as follows: 3 min of initial denaturation at 95 °C, 50 cycles of denaturation at 95 °C for 8 s, annealing at 60 °C for 60 s, followed by a final melting curve program. The mean cycle threshold value (*C*_t_) obtained from triplicate samples was used to calculate gene expression levels. PCR products were normalized to the level of glyceraldehyde 3-phosphate dehydrogenase (GAPDH) for each reaction. Relative gene expression levels are presented as the mean ± standard deviation from three independent experiments.

## Results

### Isolation of DMSCs with surface markers CD51/CD140α, CD271, and STRO-1/CD146 from DPCs

**Figure 1:**
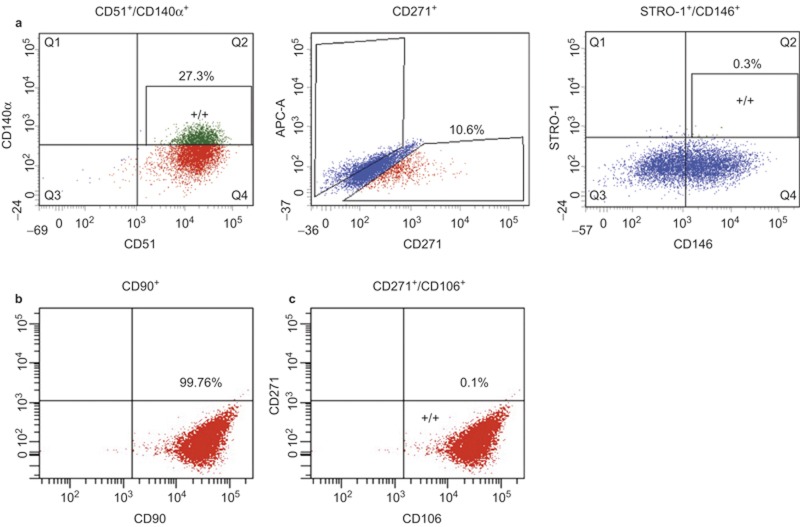
**The expression profiles of stem cell surface markers in human primary cells from DPs determined by FACS.** (**a**) CD51/CD140α, CD271, and STRO-1/CD146; (**b**) CD90; (**c**) CD271/CD106. Cells were isolated from dental pulp of adult third molars and stained with antibodies.

Many available MSC surface markers fail to represent MSCs *in vivo* as markers are not homogenously expressed across cultures.^16^ Recent studies showed that specific combinations of surface markers including CD51/CD140α and CD271/CD90/CD106 isolated highly enriched clonogenic cells from human bone marrow, respectively.^17,18^ In addition, STRO-1/CD146 combination has successfully characterized DMSCs from dental tissues such as periodontal ligament and apical papilla.^19,20^ However, whether these verified combination of surface markers are capable of isolating multipotent and self-renewing progenitor cells from human DP remains to be investigated. Using two surface combination of CD51/CD140α or STRO-1/CD146, FACS revealed that 27.3% of DPCs were CD51^+^/CD140α^+^ and 0.3% of DPCs were STRO-1^+^/CD146^+^ (Figure 1a). Interestingly, we found that the majority (99%) of DPCs expressed CD90 (Figure 1b), indicating that CD90 might not be useful. The combination of CD271/CD106 yielded extremely few positive cells (Figure 1c). Therefore, we decided to use single CD271 marker to isolate MSCs from DPCs and found that 10.6% of DPCs were CD271^+^.

### Isolated DMSCs exhibited differential odontogenic potential depending on surface markers

**Figure 2:**
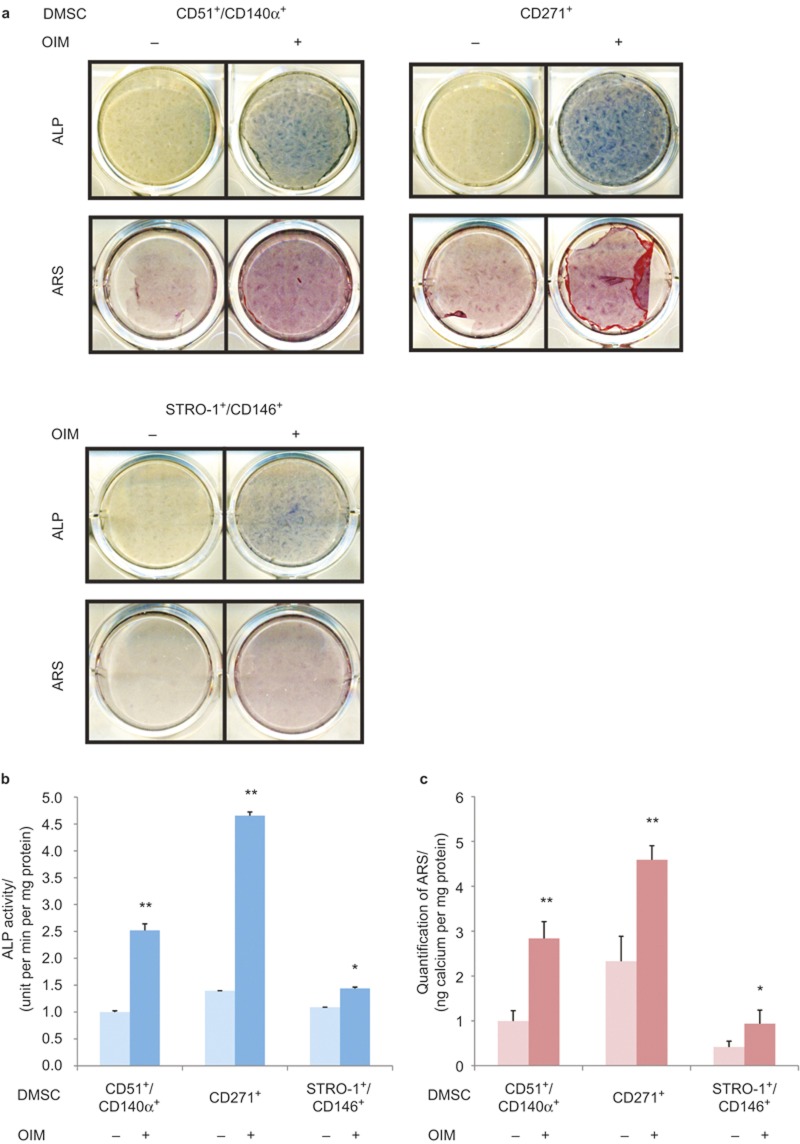
**Odontogenic differentiation of isolated CD51^+^/CD140α^+^, CD271^+^, and STRO-1/CD146^+^ DMSCs.** (**a**) ALP staining and Alizarin red staining after these cells were induced to differentiate into odontogenic lineages for 7 days and 14 days, respectively. (**b**) Quantification of ALP activity. (**c**) Quantification of ARS. Values were normalized to non-induced CD51^+^/CD140α^+^ DMSCs. *P < 0.05, **P < 0.001. ALP, alkaline phosphatase; ARS, Alizarin red stain; DMSC, dental mesenchymal stem cell; OIM, odontogenic induction medium.

To evaluate and compare differentiation capacity, isolated CD51^+^/CD140α^+^, CD271^+^, and STRO-1^+^/CD146^+^, DMSCs were induced to undergo odontogenic differentiation. All three types of isolated DMSCs exhibited the capacity to differentiate into odontogenic lineage as demonstrated by ALP staining on the seventh day (Figure 2a). Quantification of ALP activity revealed the most significant increase of ∼5 folds in induced CD271^+^ DMSCs compared to non-induced CD51^+^/CD140α^+^. Induced CD51^+^/CD140α^+^ DMSCs exhibited 2.5-fold increase in ALP activity while induced STRO-1^+^/CD146^+^ DMSCs showed 1.25-fold increase (Figure 2b). All three isolated DMSC groups had formation of mineralized nodules after prolonged treatment with odontogenic induction media for 14 days as demonstrated by ARS (Figure 2a). The quantification of ARS also showed significant mineralization potential in CD271^+^ DMSCs (4.5-fold) followed by CD51^+^/CD140α^+^ DMSCs (2.75 fold) and STRO-1^+^/CD146^+^ DMSCs (1-fold; Figure 2c).

**Figure 3:**
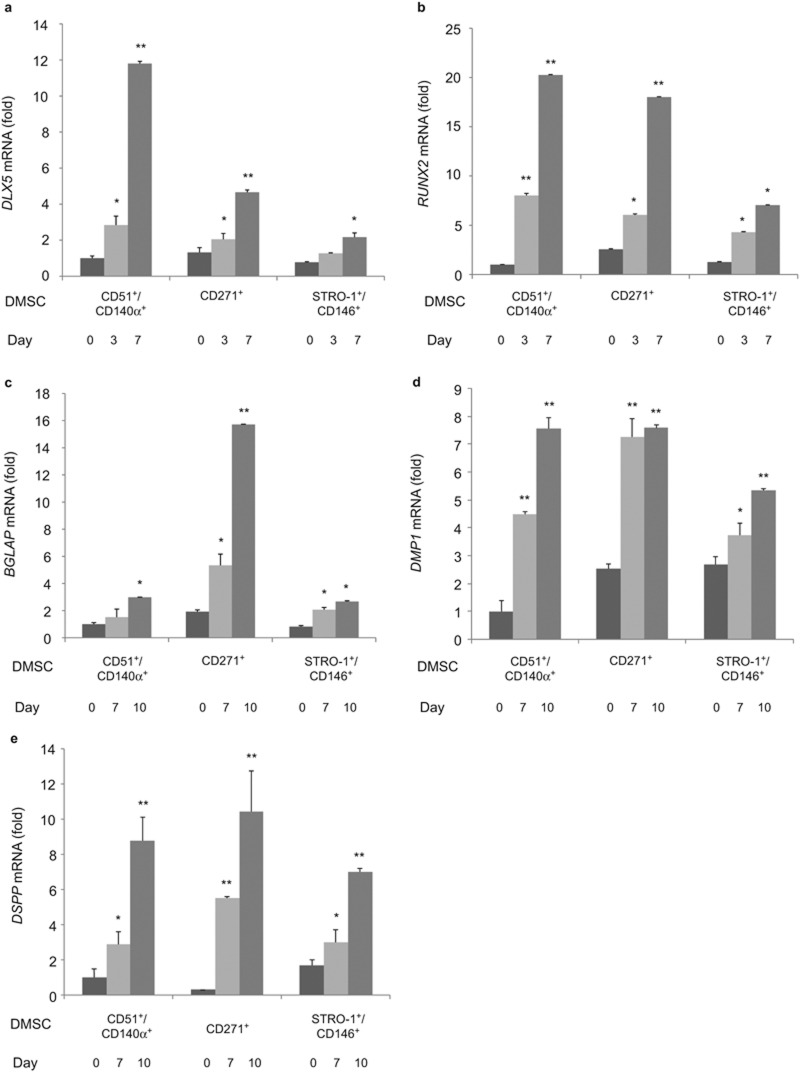
**mRNA expression of odontogenic-genes after 0, 3, 7, and 10 days of odontogenic induction in CD51^+^/CD140α^+^, CD271^+^, and STRO-1^+^/CD146^+^ DMSCs.** (**a**) *DLX5*, (**b**) *RUNX2*, (**c**) *BGLAP*, (**d**) *DMP1*, and (**e**) *DSPP*. Values were normalized to CD51^+^/CD140α^+^ DMSCs at day 0. *P < 0.05, **P < 0.001. DMSC, dental mesenchymal stem cell.

Using these isolated DMSCs, we further confirmed their odontogenic potential by examining mRNA expression of several odontogenic marker genes including *DLX5*, *RUNX2*, *BGLAP*, *DMP1*, and *DSPP* at different time points: 0, 3, 7, and 10 days after odontogenic induction. Consistent with ALP and ARS results, odontogenic marker gene expression was upregulated for all three subsets of DMSCs. In particular, marked increase of *DLX5* expression was found in CD51^+^/CD140α^+^ DMSCs at day 7 of induction compared to CD271^+^ and STRO-1^+^/CD146^+^ DMSCs (Figure 3a). The expression of *RUNX2* in both CD51^+^/CD140α^+^ and CD271^+^ DMSCs was more significantly upregulated in a time-dependent manner at day 3 and day 7 of induction (Figure 3b) compared to STRO-1^+^/CD146^+^ DMSCs. Significant *BGLAP* expression induction was found in CD271^+^ DMSCs at day 10 of odontogenic induction (Figure 3c). Lastly, in all three isolated DMSC populations, *DMP1* and *DSPP* expression were significantly upregulated at day 10 of induction (Figure 3d and 3e).

Collectively, these data suggested that homogenous populations of DMSCs isolated using FACS with different surface marker combinations are capable of differentiating into odontogenic lineages.

### Isolated DMSCs exhibited differential chondrogenic potential depending on surface markers

**Figure 4:**
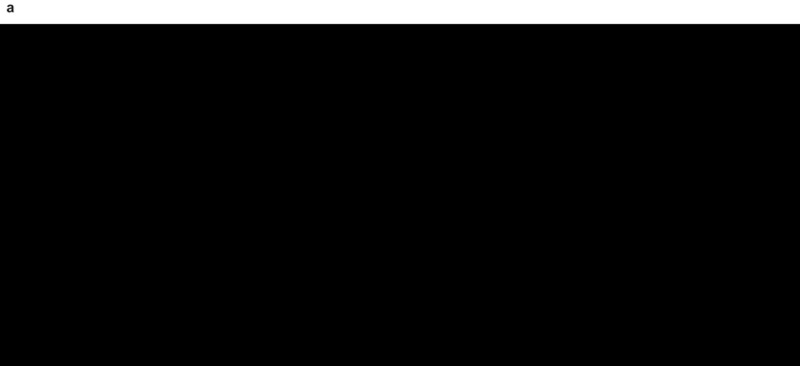
**Chondrogenic differentiation of CD51^+^/CD140α^+^, CD271^+^, and STRO-1^+^/CD146^+^ DMSCs.** (**a**) Alcian blue staining after cells were induced to differentiate into chondrogenic lineages for 28 days. (**b**) Quantification of Alcian blue. (**c**) mRNA expression of *Sox9* after 0 and 28 days of chondrogenic induction. Values were normalized to CD51^+^/CD140α^+^ DMSCs at day 0. *P < 0.05, **P < 0.001. CMI, chondrogenic induction medium; DMSC, dental mesenchymal stem cell.

We further evaluated and compared chondrogenic differentiation capacity of isolated DMSCs under chondrogenic conditions. All three isolated DMSC groups showed the presence of glycosaminogylcans as demonstrated by Alcian blue staining after prolonged treatment with chondrogenic induction media for 28 days (Figure 4a). The quantification of Alcian blue staining exhibited the greatest chondrogenic differentiation in CD271^+^ DMSCs, followed by STRO-1^+^/CD146^+^ DMSCs and CD51^+^/CD140α^+^ (Figure 4b). mRNA expression of *Sox9*, the master gene of chondrogenic differentiation, was significantly upregulated confirming their chondrogenic potential in all three populations of isolated DMSCs (Figure 4c)

## Discussion

While numerous stem cell surface markers have been routinely used to identify putative MSCs, specific markers for successful isolation of DMSCs that lead to potent differentiation into multiple lineages are lacking.^10,13^ In this study, we systematically used three different sets of surface markers (CD51/CD140α, CD271, and STRO-1/CD146) to isolate putative stem cell populations from primary DP cultures and compared their differentiation capacity into odontogenic and chondrogenic lineages. CD51/CD140α and CD271 have been previously used to isolate neural crest cell progenitors while STRO-1/CD146 combination has been used to isolate MSCs localized to blood vessels.^17,19,21,22^ In addition, the utilization of CD51/CD140α combination for DMSC isolation is not known. Our results demonstrated that although all three combinations of surface markers were able to isolate DMSCs from DP with varying magnitudes of differentiation capabilities, CD271 was the best single marker for isolating the DMSC progenitor population with greatest differentiation potential. Adipogenic potential was evaluated but found to be minimal for all three subsets of DMSCs (data not shown).

About 10.6% of primary culture DPCs were positive for CD271 (Figure 1). CD271, also known as the p75 neurotrophin receptor (p75^NTR^ or low-affinity nerve growth factor), has been described as one of the most reliable surface markers for isolation of putative BMSCs and identified as a *bona fide* neural crest stem cell marker.^23,24,25,26^ On the contrary to our studies, CD271^+^ cells from CD44^+^/CD90^+^ MSCs of human deciduous DPs could not differentiate into osteoblasts and adipocytes.^21^ Currently, the reasons for these conflicting results are unknown. It could be due to the discrepancy in tissue sources for MSC isolation. However, CD271 was previously used in combination with THY-1/VCAM-1 in BMSCs.^18^ Therefore, we first examined THY-1 (CD90) in characterizing DPCs and our preliminary FACS data showed 90% of DPCs expressed THY-1 (CD90), suggesting its lack of specificity for isolating DMSCs. We further characterized DPCs with a combination of CD271 and VCAM-1 (CD106) and found that 0.1% of DPCs expressed both CD271 and VCAM-1 markers. For these reasons, the isolation of DPSCs in this study was performed with a single marker, CD271. Our study showed that CD271^+^ DMSCs exhibited the most significant odontogenic differentiation potential with high ALP activity, mineralization capacity, and upregulated expression of *DLX5*, *RUNX2*, *BGLAP*, *DMP1*, and *DSPP* (Figures 2 and 3). Similarly, during chondrogenic lineage differentiation, CD271^+^ DMSCs exhibited the greatest chondrogenic potential demonstrated by increased Alcian blue staining and *SOX9* expression levels. Therefore, CD271 can be identified as a single specific surface marker for isolating putative DMSCs that are highly multipotent.

The use of CD51/CD140α to isolate DMSCs from DP has not been previously studied. CD51 and CD140α are highly expressed in human Nestin^+^ sphere forming MSCs, which are capable of hematopoetic progenitor cell expansion.^17^ Nestin is mainly expressed in early stages of the central nervous system and muscle development.^27,28^ CD51 labels the integrin V alpha subunit, which can form heterodimers with at least five distinct beta subunits.^29,30^ CD140α, platelet-derived growth factor receptor (PDGFR) α is a tyrosine kinase receptor and found to be expressed on mesenchymal-derived cells. ^31^ As such, the use of CD51/CD140α identified a large subset of perivascular Nestin^+^ cells highly enriched for MSCs in both human and mouse bone marrow *in vivo*.^17^ Our results demonstrated that CD51 and CD140α markers are also useful for isolating DMSCs that exhibit multilineage potential. (Figures 2, 3, 4) On average, 27.3% of DPCs were positive for both CD51 and CD140α, providing the largest proportion of DPCs among the three surface marker combinations in this study (Figure 1).

STRO-1 and CD146 are considered to be early MSC surface markers that have been identified in BMSCs and are derived from perivascular cells.^32^ STRO-1 was initially associated with the identification of osteogenic precursors isolated from bone marrow and later described as a promising marker for MSCs.^33^ However, it was suggested that the use of STRO-1 as a single marker for MSC isolation is limited; as it is not universally expressed in all types of MSCs and it can gradually decline with increasing culture passages.^5,34,35^ In this study, CD146 was used with STRO-1 to address these STRO-1-related concerns. The lowest proportion of DPCs, 0.3%, was STRO-1^+^/CD146^+^ (Figure 1), making it challenging to expand the STRO-1^+^/CD146^+^ DMSCs for further use. The proportion of STRO-1^+^/CD146^+^ cells was significantly smaller in these DPCs compared to periodontal ligament cells (2.6%) and apical papilla cells (13.4%).^19,20^ While ALP activity and mineralization capacity were increased in induced STRO-1^+^/CD146^+^ DMSCs compared to non-induced STRO-1^+^/CD146^+^ DMSCs, the enhancement was not as significant as CD271^+^ or CD51/CD140α DMSCs. Less significant odontogenic and chondrogenic potential was found in STRO-1^+^/CD146^+^ DMSCs compared to the other two combinations (Figures 2 and 3). Although STRO-1 is a widely utilized MSC marker, it is not the best surface marker for isolating DMSCs with high differentiation capabilities from DPCs even when combined with CD146.^22,33,35^

It is noteworthy that DP is originally derived from neural crest cells that have both ectodermal and mesenchymal components and can provide a pure MSC population.^13,36,37,38^ As some recent studies hypothesized that MSCs are neural crest derived, isolating DMSCs with surface markers of a neural crest origin such as CD51/CD140α and CD271 may be desirable.^39,40^ In line with this notion, our study indicated that CD271^+^ and CD51^+^/CD140α^+^ DMSCs exhibited more significant multipotent populations from DP compared to STRO-1^+^/CD146^+^ DMSCs. Therefore, isolation of DMSCs using surface markers of neural crest origin other than CD271 and CD51/CD140α may be important to further evaluate their potency in differentiation potential.

In conclusion, our findings demonstrated the successful isolation of homogenous populations of DMSCs from heterogenous DPCs obtained from DP with the use of specific neural surface markers and showed their ability to differentiate into odontogenic and chondrogenic lineages. Our results suggest that: (1) there are heterogeneous MSC populations in DPCs, and (2) lineage-specific differentiation potential is variable among distinct-isolated DMSC populations. Specifically, the CD51/CD140α marker isolated the largest population of DMSCs (27.3%) and showed relatively strong odontogenicand chondrogenic potential, while STRO-1^+^/CD146^+^ DMSCs were more limited in number (0.3%) and differentiation potential. The most effective marker was CD271, which isolated a relatively large population of DMSCs (10.6%) and had the strongest odontogenic and chondrogenic potential. Further studies are needed to validate whether these isolated cells may differentiate into functionally different lineages *in vivo*.
