# Author Correction: Single CD271 marker isolates mesenchymal stem cells from human dental pulp

**DOI:** 10.1038/s41368-018-0042-x

**Published:** 2019-01-02

**Authors:** Ruth Alvarez, Hye-Lim Lee, Christine Hong, Cun-Yu Wang

**Affiliations:** 1 Division of Oral Biology and Medicine, School of Dentistry, University of California at Los Angeles, Los Angeles, USA; 2 Section of Orthodontics, School of Dentistry, University of California at Los Angeles, Los Angeles, USA; 3 Department of Bioengineering, Henry Samueli School of Engineering and Applied Science, University of California at Los Angeles, Los Angeles, USA

Correction to: *International Journal of Oral Science* (2015) **7**, 205–212; 10.1038/ijos.2015.29; published 18 September 2015

In the original version of this Article, Fig. 1c was inadvertently assembled with a duplicate of Fig. 1b. The correct image for Fig. 1c is showed below. This does not affect the conclusions of the study. We sincerely apologize for any inconvenience this may have caused our readers.

**Fig. 1:**
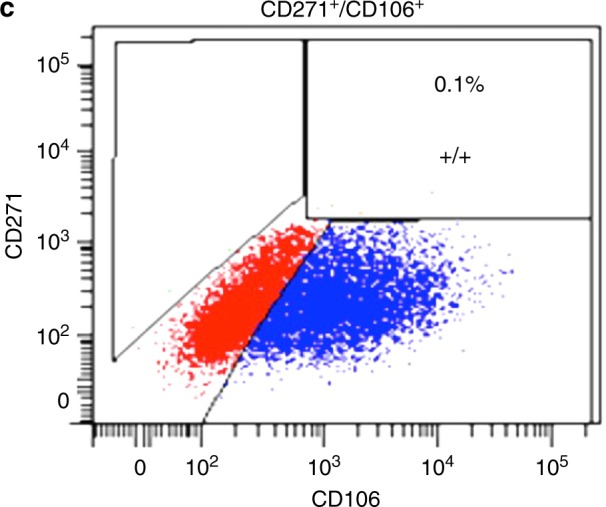
The expression profiles of stem cell surface markers in human primary cells from DPs determined by FACS

